# Construction of Recombinant HVT Expressing PmpD, and Immunological Evaluation against *Chlamydia psittaci* and Marek’s Disease Virus

**DOI:** 10.1371/journal.pone.0124992

**Published:** 2015-04-20

**Authors:** Shanshan Liu, Wei Sun, Jun Chu, Xiufen Huang, Zongxue Wu, Minxin Yan, Qiang Zhang, Peng Zhao, Joseph U. Igietseme, Carolyn M. Black, Cheng He, Yongqing Li

**Affiliations:** 1 Key Lab of Animal Epidemiology and Zoonosis of Ministry of Agriculture, College of Veterinary Medicine, China Agricultural University,Beijing 100193, China; 2 Institute of Animal Husbandry and Veterinary Medicine, Beijing Academy of Agricultural and Forestry Sciences, Beijing 100097, China; 3 National Center for Emerging and Zoonotic Infectious Diseases, CDC, Atlanta, Georgia, 30333, United States of America; The University of Melbourne, AUSTRALIA

## Abstract

*Chlamydia psittaci* (*C*. *psittaci*) is an obligate intracellular zoonotic pathogen that can be transmitted to humans from birds. No efficacious commercial vaccine is available for clearing chlamydial infection due to lack of potential vaccine candidates and effective delivery vehicles. Herpesvirus of turkeys (HVT) is an efficacious commercially available vaccine against Marek’s Disease virus (MDV). In this study, a recombinant HVT-delivered vaccine against *C*. *psittaci* and Marek’s disease was developed and examined. The 5'-terminus of *pmpD* gene (*pmpD*-N) encoding the N-terminal fragment of polymorphic membrane protein D of *C*. *psittaci* was inserted into a nonessential region of HVT genome using reverse genetics based on an infectious bacterial artificial chromosome (BAC) clone of HVT. The recombinant virus (rHVT-*pmpD*-N) was recovered from primary chicken embryo fibroblast (CEF) cells by transfection of modified HVT BAC DNA containing the *pmpD*-N gene. The rHVT-*pmpD*-N construct was confirmed to express PmpD-N by immunoblot and immunofluorescence. The rHVT-*pmpD*-N was stable during 20 passages *in vitro*. The growth kinetics of rHVT-*pmpD*-N was comparable to that of parental HVT *in vitro* and *in vivo*. One-day-old SPF chickens inoculated subcutaneously with rHVT-*pmpD*-N displayed increased PmpD-specific antibody levels and a vigorous PmpD-specific lymphocyte proliferation response using HVT vector or CEF cells as control. Furthermore, the percentage of CD4+ cells was significantly elevated in rHVT-*pmpD*-N-immunized birds as compared to the parental HVT. All chickens vaccinated with rHVT-*pmpD*-N or parental HVT were protected completely against challenge with a very virulent strain of Marek’s Disease virus (MDV) RB-1B. Post challenge with *C*. *psittaci* CB7 strain, a significant decrease in respiratory distress, lesions and *Chlamydia* load was found in the rHVT-*pmpD*-N-vaccinated group compared to the parental HVT. In conclusion, our study suggests that the rHVT-*pmpD*-N live vaccine may be viable as a candidate dual vaccine that provides protection against both very virulent MDV and *C*. *psittaci*.

## Introduction

The obligate intracellular Gram-negative bacterium *Chlamydia psittaci* causes systemic disease in psittacine birds, domestic poultry, and wild fowl [1]. In poultry, the pathogen often induces a disease called ornithosis, generally referred to as chlamydiosis in other bird species. At present, six genotypes of *C*. *psittaci* (A-F) have been considered endemic in poultry and other birds [2, 3]. Avian *C*. *psittaci* strains have been reported in many countries, such as Austria [4], France [5], China [6] and Germany [7]. Moreover, high seroprevalence rates were identified in Belgian and Northern French chicken farms using an indirect ELISA, with seropositivity rates of 96% and 90% respectively in broiler and layer flocks [8]. Importantly, *C*. *psittaci* is also a zoonotic pathogen capable of causing pneumonia, encephalitis, endocarditis and even death in humans [9]. Thus avian chlamydiosis is not only associated with severe economic losses in the poultry industry, but also with a potentially serious health hazard to humans who come in close contact with infected birds [10, 11].

In previous reports, DNA vaccines expressing recombinant *OmpA* gene (MOMP) [12–17] and *ompA*-based adenovirus vaccines [9] have shown promise in protection against *C*. *psittaci* infection. However, the protection elicited by MOMP-based vaccines was only partial and homotypic [18]. Recent studies have shown that members of the autotransported polymorphic membrane protein (Pmp) family of *Chlamydia* spp. are highly immunogenic vaccine candidates [19]. Among the Pmps, PmpD is most conserved by sequence and is the target of broadly cross-reactive neutralizing antibodies. PmpD can elicit early immune-mediated neutralization of an ongoing chlamydial infection [18, 20]. Therefore, PmpD is an attractive vaccine candidate. The N-terminal fragment of PmpD (PmpD-N) is translocated to the surface of the bacterium where it may non-covalently bind to other components of the outer membrane. Thus PmpD-N-specific neutralizing antibody may provide humoral immune protection against early infection [20]. In our pilot study [21], chickens immunized twice with recombinant PmpD-N expressed in *E*. *coli* were partially protected post-challenge with *C*. *psittaci*. Therefore, PmpD-N is a suitable candidate antigen toward the development of a *C*. *psittaci* vaccine.

Herpesvirus of turkeys (HVT) is an efficacious commercial available vaccine against Marek’s Disease virus (MDV) in chickens, which is considered one of the most potent delivery vectors for polyvalent live vaccines [22]. A HVT vector-based vaccine is able to induce cellular and humoral immunity by delivering specific antigens to the surface of the cell [23]. This property is well-suited for the development of a vaccine to prevent infection by obligate intracellular pathogens such as *C*. *psittaci* [15]. Additionally, this vaccine may also induce lifetime protection against MDV and other related viruses, a significant benefit as a single vaccination is more practical and causes less stress to the birds. Furthermore, HVT-based recombinant vaccines expressing antigens using the bacterial artificial chromosome (BAC) system are stable both *in vitro* and *in vivo* [24].

In this study, we generated a recombinant HVT vaccine expressing the N-terminal fragment of PmpD of *C*. *psittaci* strain CB7 (rHVT-*pmpD*-N) by modifying the HVT genome within a BAC. Then, the immunogenicity and protective efficacy of the vaccine were evaluated in SPF chickens.

## Materials and Methods

All animals were handled in strict accordance with the Regulations for the Administration of Affairs Concerning Experimental Animals of the State Council of the People’s Republic of China. The protocol was approved by the Committee on Experimental Animal Management of China Agricultural University.

### Cells, *C*. *psittaci* strain and virus

Buffalo Green Monkey (BGM) cells [25] used for the propagation of *C*. *psittaci* stocks were donated by Professor Chengming Wang, Yangzhou University, China. Primary chicken embryo fibroblast (CEF) cells were prepared from 10-day-old specific-pathogen-free (SPF) embryos (Vital Merial Experimental Animal Co., Ltd, Beijing, China). The mild-virulence *C*. *psittaci* strain CB7 (genotype A) originally isolated from a wild bird in Wuhan, China [26] was purchased from the China Institute of Veterinary Drug Control (IVDC, Beijing, China), inoculated into BGM monolayers and titrated according to standard protocols [27]. The standardized aliquots were frozen at -80°C until DNA extraction or use in challenge studies. *C*. *psittaci* CB7 strain cause typical chlamydiosis lesions in SPF chickens comparable to that of *C*. *psittaci* 6BC strain in our previous study [28]. The highly oncogenic MD RB-1B strain originated from Dr. KA Schat at Cornell University, USA [29] was used in the MDV challenge study.

### Construction of recombinant HVT-BAC expressing *pmpD*-N gene

Primers specific for *pmpD*-N shown in Table 1 were designed using Oligo 7 (Molecular Biology Insights, USA.) based on the gene sequence in GenBank (accession no. L25436). The genomic DNA of the CB7 strain was extracted by using DNA Mini Kit (Qiagen Ltd., Crawley, UK) according to the manufacturer’s instructions. The *pmpD*-N gene fragment was amplified by PCR using primers *pmpD*F and *pmpD*R, and the 1191 bp amplicon was cloned into the *Kpn*I and *Eco*RV sites of pVAX1 (Invitrogen, Carlsbad, CA). The correct orientation of the insert was confirmed by nucleotide sequencing. The expression cassette with the CMV promoter at the 5′ end and the BGH polyadenylation (poly A) site at the 3′ end was amplified using PCR (primers shown in Table 1). The amplified expression cassette was cloned into the pGEM-T vector (Promega, Madison, WI) to construct HVT transfer vector (pHVT-UL45-46) in *Pac*I site, which was flanked by 1.3 kb UL45 and 1.4 kb UL46 gene sequences of HVT. The procedures used in the construction of a recombinant HVT BAC containing the *pmpD*-N expression cassette were described as previously [24, 30]. First, the *galK* report gene was inserted to the UL45 and UL46 region of the HVT BAC by homologous recombination and positive selection. Then, the *pmpD*-N gene expression cassette linearized with *Pvu*II and *Nru*I restriction enzymes was transformed into *E*. *coli* SW105 to substitute the *galK* gene by homologous recombination and negative selection. Consequently, positive colonies were isolated on 2-deoxy-galactose and chloramphenicol-containing minimal medium plates with glycerol as the carbon source. The recombinant HVT-BAC was identified by PCR. The integrity of the *pmpD-*N gene sequence was confirmed using *pmpD*-N and HVT-specific primers for sequencing.

**Table 1:** Oligonucleotide primers used in the research.

Target sequence	Primer name	Primer sequence(5'-3')	Amplicon size (bp)	Tm (°C)
*pmpD*-N gene	*pmpD*F	CGGGGTACCGCCGCCACCATGGGATCCAATGTGTTGATTTCTGGAA (*Kpn*I site underlined)	1191	60
	*pmpD*R	AAAGATATCTCAAACAGCCCCACCTGTAGGAGCA (*Eco*RV site underlined)		
expression cassette	cassetteF	GTTCCGCGTTAATTAACTTACGGTA (*Pac*I site underlined)	2149	55
	cassetteR	TGTTAATTAAGGTTCGCTTGCTGT (*Pac*I site underlined)		
sorf1 gene	HVTF	AGTCTCGAGCGTGGACAGAT	175	60
	HVTR	CCAAACGTCCGTAGACGAAT		

### Generation of recombinant HVT-*pmpD*-N

The recombinant HVT was generated as described previously [24]. Briefly, the recombinant HVT-BAC DNA (1 μg) was transfected into CEF cells using lipofectamine 2000 transfection reagent (Invitrogen, Carlsbad, CA), and the transfected cells were maintained in M199 with 1% FBS for 6 h. The cells cultured at 37°C in 5% CO2 were harvested on day 4 post-transfection when CPE was observed. The recombinant virus was designated as rHVT-*pmpD*-N.

### Plaque assays and one-step growth kinetics

The plaque size, morphology and plaque-forming unit (PFU) of rHVT-*pmpD*-N were compared with those of the same passage of parental HVT by the immunohistochemical assay as described previously [24]. The growth rates of the rHVT-*pmpD*-N were studied on CEF cells by calculating the gene copy number at various time points as described previously with some modifications [31]. Briefly, 400 PFU of rHVT-*pmpD*-N and parental HVT vector were simultaneously inoculated onto CEF cells in 6-well plates. At 12, 24, 48, 72, 96 and 120 h post-infection, virus-infected CEF cells were harvested. DNA was prepared by phenol extraction [32], and was diluted to the concentration of 1 μg/mL. HVT genome copies were quantified by adding 5 μL the extracted DNA using conventional SYBR Green real-time PCR method for absolute quantification. Primers (Table 1) were designed according to the sorf1 region of HVT FC126 (AF282130), and synthesized by a commercial company (Sangon, Shanghai, China).

### Identification of the rHVT-*pmpD*-N using immunoblot and immunofluorescence

Immunoblot analysis was carried out as described previously [24]. Briefly, 3000 PFU of rHVT-*pmpD*-N and parental HVT were used to infect CEF cells seeded in T25 flasks. The cells were washed twice with phosphate-buffered saline (PBS) when cytopathic effect (CPE) occurred. Lysates of cells were subjected to 12% SDS-PAGE and electro-bloted to polyvinylidene fluoride (PVDF) membranes, followed by 0.25% trypsin for 2 min. Membranes were incubated with the *C*. *psittaci* strain 6BC-specific polyclonal antibodies (diluted 1:100) previously prepared by our own lab (see Text A in S1 Dataset), and then reacted with horseradish peroxidase-labelled goat-anti-chicken IgG (1:4000) (Sigma-Aldrich, Shanghai, China).

The expression and distribution of PmpD-N was determined in rHVT-*pmpD*-N-infected cells by immunofluorescence [24]. First, CEF cells were infected with 3000 PFU rHVT-*pmpD*-N and parental HVT, respectively. The cells were probed with mouse anti-PmpD-N polyclonal serum of *C*. *psittaci* (previously prepared in our own lab, see Text B in S1 Dataset) and anti-HVT polyclonal serum (IVDC, Beijing, China) at a dilution of 1:100, and then overlaid with a mixture of goat anti-mouse IgG labeled with Alexa Fluor 488 (Invitrogen, Carlsbad, CA) and goat anti-chicken IgY labeled with Alexa Fluor 568 (1:600 dilution) (Invitrogen, Carlsbad, CA). Moreover, cell nuclei were stained with 1:10,000 dilution of 4,6 diamidino-2-phenylindole (DAPI) for 1 min or longer. Finally, the rinsed cover slips were mounted with Vectashield mounting medium (Vector Laboratories, Burlingame, CA) and examined using a confocal microscope (Nikon, Tokyo, Japan).

### Stability test of rHVT-*pmpD*-N

The rHVT-*pmpD*-N vaccine was grown in CEF cells for 20 serial passages. After every 5 passages, the presence of the *pmpD*-N sequence was detected by PCR using specific primers while the expression of PmpD-N was determined using indirect immunofluorescence as described above.

### Immunization and challenge experiments

In this study, 65 one-day-old SPF chickens (Vital Merial Experimental Animal Co., Ltd, Beijing, China) were randomly divided into three groups and reared in negative pressure isolators individually in order to reduce co-infection. Twenty-five chickens in rHVT-*pmpD*-N group were immunized by 8000 PFU of rHVT-*pmpD*-N, while 20 SPF chickens in parental HVT group were given with 8000 PFU of parental HVT (recovered from BAC-clone). A total of 20 chickens in the CEF control group were inoculated with uninfected CEF cells (1.3 × 10^5^ cells per dose) as the negative control. The actual dose of vaccination was calculated by titrating the remaining parental HVT and recombinant HVT in CEF cells after immunization. All chickens were inoculated subcutaneously at the back of the neck.

Seven days after immunization, 10 chickens in each of the three groups (CEF control group, parental HVT group and rHVT-*pmpD*-N group) were kept in separate isolators to inoculate intraperitoneally with a very virulent strain of MDV RB-1B (1000 PFU per chicken) and then monitored daily for clinical signs. Mortality was evaluated for 60 days. Finally, the chickens were examined for MDV-derived lesions, such as presence of tumor and organ lesions [24]. The remaining 10 or 15 chickens in each group were not challenged with RB-1B.

On day 36, those chickens in each group which had not been previously challenged with RB-1B, were now taken to a different unit to infect intra-tracheally with 0.1 ml of 5×10^8.5^ TCID_50_ *C*. *psittaci* strain CB7 as previously described [28]. All chickens were observed daily for clinical signs and morbidity, and euthanized on day 4 post-challenge. Gross lesions, bacterial shedding and bacterial load in tissues were determined as described previously with some modifications [12–17]. Before sacrifice, pharyngeal swabs were collected and inoculated to BGM cells to determine the shedding of *C*. *psittaci* by immunofluorescence (Dako, Cambridge, UK). The number of chlamydial inclusions was counted in five randomly selected microscopic fields at the amplification of 400×. A score from 0 to 3 was given for each swab individually. Score 0 means that there were no chlamydial inclusions. Scores 1, 2 and 3 were given when a mean of 1–2, 3–5 and >6 inclusions were observed, respectively [12–17]. Lungs of chickens in the three groups were aseptically isolated, and the tissue suspensions were minced and prepared as described previously [33]. Tissue suspensions cultured in BGM cells were used to evaluate the presence of *C*. *psittaci*, and chlamydial infectivity was calculated on the basis of the dilution titer of the original inoculum.

### Detection of rHVT-*pmpD*-N replication in vaccinated chickens

The replication of rHVT-*pmpD*-N in vaccinated chickens was measured in peripheral blood leukocytes (PBLs). Blood samples (0.5 mL per chicken) from vein puncture were collected and transferred immediately into aseptic capped tubes with sodium heparin, then diluted with equal volume of Hanks’ solution and carefully layered on the surface of 1ml lymphocyte separation medium (Sigma, USA). After centrifugation at 3000 rpm for 20 min, PBLs were collected from the interface of the Histopaque and serum, and then added to Hanks’ solution [34]. DNA was extracted from PBLs as described above, and was diluted to the concentration of 15 μg/mL. HVT genome copies were quantified by adding 5 μL the extracted DNA using SYBR Green real-time PCR as described above.

### Humoral response

Blood samples were collected weekly from all chickens after immunization. Anti-PmpD-N antibody levels were measured using PmpD-N ELISA established by an in-house kit. Briefly, the indirect ELISA was performed using the following procedure. Ninety-six well ELISA plates were coated with 100 μl/well (4 μg/ml) of purified PmpD-N protein diluted in 0.05 M bicarbonate/carbonate buffer (pH 9.6) at 4°C overnight. Subsequently, the plates were washed 4 times with PBS containing 0.05% Tween 20 (PBST) to remove unbound antigen, and then the wells were blocked at 37°C for 2 h using 200 μl of blocking buffer (PBS containing 5% skim milk), followed by another washing step. 100 μl of sera diluted 1:50 in PBS was added into each well and incubated for 1 h at 37°C. Horseradish peroxidase–conjugated goat anti-chicken antibodies were diluted 1:10000 in PBS, and 100 μl were added into each well. After incubation and washing steps were performed as described above, the colorimetric reaction was started by the addition of 100 μl/well 3,3′,5,5′-tetramethylbenzidine (TMB) (Qiagen, Stuttgart, Germany) at 37°C for 10 min, and 2M H_2_SO_4_ was used to stop the color development. The plates were read using a universal Microplate Reader (Thermo Life Sciences, Shanghai, China) at 450 nm/630 nm. A serum sample was considered positive when its OD value exceeded 0.045.

### Lymphocyte proliferative responses and analysis of T-lymphocyte subsets

Before challenge, PBLs (*n* = 5 for each group) were prepared, and lymphocyte proliferative responses and T-lymphocyte subsets in peripheral blood were evaluated. As for antigen proliferation, 400 PFU of inactivated rHVT-*pmpD*-N were added to individual wells, while Concanavalin A (ConA) (Sigma-Aldrich, Shanghai, China) was added at 5 μg/well as a positive control and medium was used as a background control. All experiments were performed in triplicate. The plates were incubated at 37°C in 5% CO2. Con A- or antigen-induced proliferation was pulsed with 0.25 μCi of [^3^H] thymidine (Isotope Corporation, Beijing, China) per well for 16 h. The cells were harvested on to glass microfiber filters (Whatman, Kent, UK), and activity was counted in a HIDE X 300SL Automatic TDCR liquid scintillation counter(Nature Gene life Sciences, Beijing, China).

T-lymphocyte subsets in peripheral blood (*n* = 5 for each group) were evaluated by flow cytometric analysis. Collected cells (1 × 10^6^ in 1 mL) from PBLs were centrifuged again, and the pellets were re-suspended with 200μL PBS. After addition of 2μL of a mixture of SPRD-conjugated mouse-anti-chicken CD3+, FITC-conjugated mouse-anti-chicken CD4+ and RPE-conjugated mouse-anti-chicken CD8+ (Southern Biotech, Birmingham, AL), the mixture was incubated at 4°C for 20 min in the dark. After centrifugation (3000 rpm at 4°C for 10 min), the pelleted cells were washed with PBS, and re-suspended in 0.5 mL PBS with fetal bovine serum [35]. The percentages of CD4+ and CD8+ cells were assessed by analyzing 20,000 living cells using FACS Canto flow cytometry (Becton Dickinson, Franklin Lakes, USA). Dead cells were eliminated based on their light scatter characteristics.

### Statistical analysis

For analysis of the protective efficacy against MDV, Chi-square test was used. For the analysis of gross lesion scores and bacterial shedding of *C*. *psittaci*, the median test was used. Other data derived from comparing experimental groups were analyzed using the one-way ANOVA. A *p*-value of less than 0.05 was considered statistically significant.

## Results

### Construction and recovery of recombinant HVT-*pmpD*-N

The *pmpD*-N gene with CMV promoter was successfully inserted into the HVT BAC region between UL45 and UL46. The recombinant HVT-*pmpD*-N BAC DNA was transfected into CEF cells. Four days after transfection, typical plaques appeared in the CEF monolayers, these plaques being similar in size and morphology to those formed by parental HVT (S1 Fig). The viral titration by staining plaques revealed no significant difference between recombinant and parental HVT.

### Expression of PmpD-N in rHVT-*pmpD*-N infected cells

**Fig 1:**
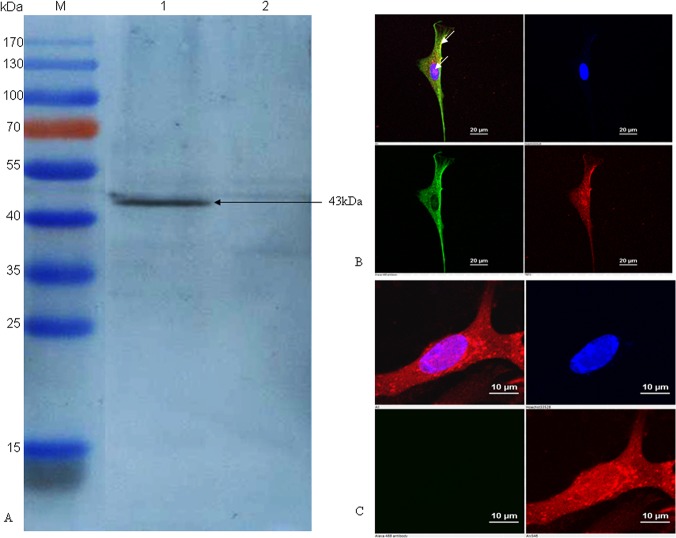
Confirmation of PmpD-N protein expression in HVT vector by immunoblotting assay and indirect immunofluorescence. (A) The PmpD-N expression in rHVT-*pmpD*-N was detected by immunoblot with *C*. *psittaci* strain 6BC-specific polyclonal antibodies. Lane M, pre-stained protein ladder; Lane 1, cell lysate post inoculation with rHVT-*pmpD*-N; Lane 2, cell lysate post inoculation with parental HVT. The black arrow indicates the approximately size of 43kDa. (B) Indirect immunofluorescence analysis of PmpD-N expression in CEF cells. CEF cells on glass coverslips were infected with rHVT-*pmpD*-N, then incubated with mouse anti-PmpD-N polyclonal antibody of *C*. *psittaci* and chicken anti-HVT polyclonal serum, and then reacted with goat anti-mouse IgG conjugated with Alexa Fluor 488 (green fluorescence, shown in the lower left panel) and goat anti-chicken IgY labelled with Alexa Fluor 568 (red fluorescence, shown in the lower right panel), respectively. Finally, cell nuclei were stained with DAPI (blue fluorescence, shown in the top right panel). The merged image is shown in the top left panel. The expression of the targeted protein is indicated by white arrows in top left panel. (C) Parental HVT control. CEF cells on glass coverslips were infected with parental HVT, and then the process of test and the panel meaning are the same as those shown in Fig 1B.

To confirm the expression of the PmpD-N protein, CEF cells infected with rHVT-*pmpD*-N were analyzed by immunoblot and immunofluorescence. A 43-kDa band corresponding to the PmpD-N polypeptide was identified with polyclonal antibodies generated against *C*. *psittaci* 6BC strain by immunoblot analysis (Fig 1A). Under immunofluorescence microscopy, PmpD-N protein was expressed in the cytoplasm and on the cell surface (Fig 1B), while HVT proteins were mainly localized in nucleus and cytoplasm (Fig 1C).

### Genetic stability of rHVT-*pmpD*-N and one-step growth kinetics

Samples were collected from every 5 passages and the genetic stability of rHVT-*pmpD*-N growing in the CEF cells was confirmed by PCR and immunofluorescence. PCR analysis confirmed that the *pmpD*-N gene was stably inserted into the vector and consistently maintained after over 20 passages (S2 Fig). Moreover, PmpD-N protein was consistently expressed in the same amount in all passages as determined by immunofluorescence (S3 Fig).

**Fig 2:**
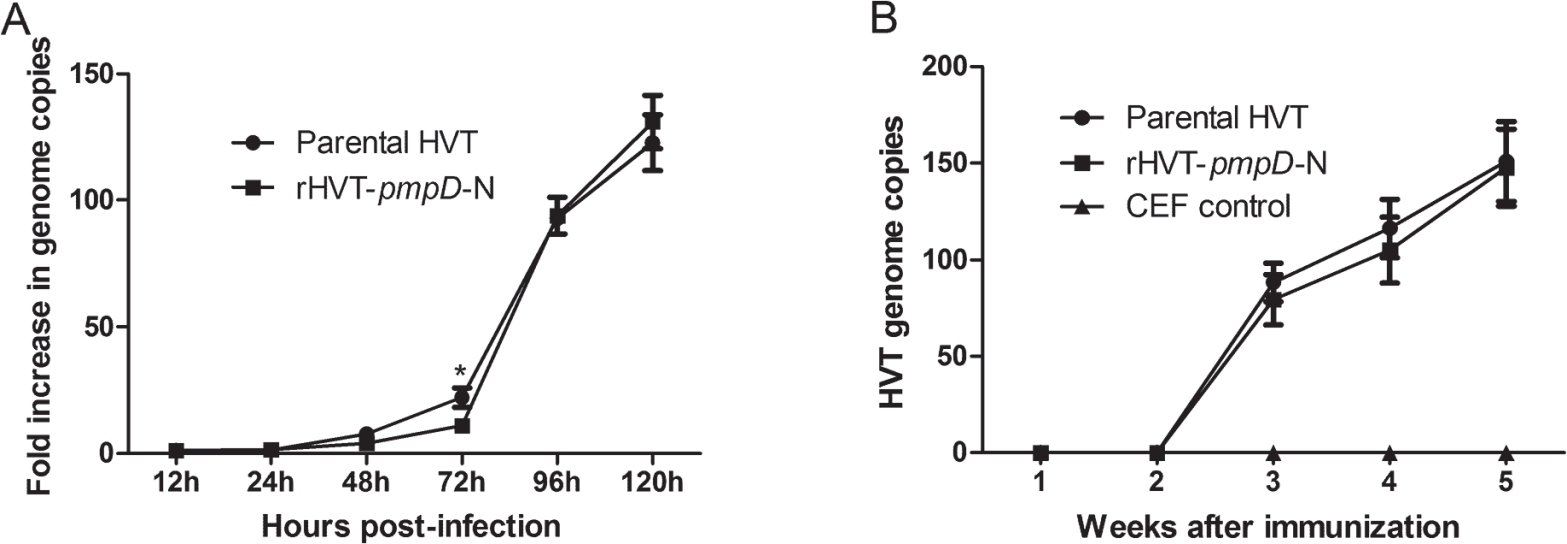
One-step growth kinetics of rHVT-*pmpD*-N and parental HVT *in vitro* and *in vivo*. (A) The virus growth was calculated as the fold increase at different time points compared with the 12^th^ h post infection. The asterisk indicates significant differences of the growth kinetics between rHVT-*pmpD*-N and parental HVT (*P*<0.05). (B) The replication kinetics of parental HVT (*n* = 10) and rHVT-*pmpD*-N (*n* = 15) in vaccinated chickens was evaluated by real time PCR in PBLs. The HVT genome copies were quantified in 75 ng extracted DNA at different time point. The values were shown as means ± standard deviation.

With respect to the growth kinetics, rHVT-*pmpD*-N was comparable to HVT vector as revealed by genome copy measurements at various times post-infection (Fig 2A). Growth rates displayed a slight increase between 24 h and 72 h, and a sharp increase from 72 h to 120 h. However, the growth rate of the parental HVT was consistently slightly higher than that of rHVT-*pmpD*-N, with a significant difference appearing only at 72 h post infection.

### Evaluation of protection post-challenge with MDV

Results of protective efficacy of the recombinant vaccine against MD are shown in Table 2. Chickens vaccinated with rHVT-*pmpD*-N and parental HVT did not show any clinical signs or histopathological lesions during 60 days after challenge with MDV. In contrast, 2 out of 10 control chickens (20%) were found dead, 3 out of 10 chickens (33.3%) had clinical signs, and all chickens were observed with typical MD lesions in the control group. Results from the parental HVT group or the rHVT-*pmpD*-N group revealed significant difference in protection as compared to the control group.

**Table 2:** Protective efficacy of rHVT-*pmpD*-N and parental HVT against challenge with very virulent MDV strain RB-1B.

Groups^a^	Tumours /total	Organ lesions/total	Clinical signs/total	Mortality/total	Protection rate (%)
rHVT-*pmpD*-N	0/10	0/10	0/10	0/10	10/10(100%)
Parental HVT	0/10	0/10	0/10	0/10	10/10(100%)
CEF control	10/10	10/10	3/10	2/10	0/10 (0%)

^a^ Twenty-five 1-day-old SPF chickens in the rHVT-*pmpD*-N group were immunized with 8000 PFUs of rHVT-*pmpD*-N, while 20 SPF chickens in parental HVT group were immunized with 8000 PFUs of parental HVT (recovered from BAC-clone). A total of 20 chickens in the CEF control group were inoculated with uninfected CEF (1.3 × 10^5^ cells per dose) as the negative control. Seven days after immunization, 10 chickens from each group were inoculated intraperitoneally with a virulent strain of MDV RB-1B (1000 PFUs/chicken) and then monitored for 60 days.

### Detection of rHVT-*pmpD*-N in vaccinated chickens

The replication kinetics of parental HVT and rHVT-*pmpD*-N in vaccinated chickens was evaluated by real time PCR in PBLs. Both viruses showed no significant differences (*p*>0.05) in replication and very similar genome copies were observed over the 5-week period following vaccination (Fig 2B).

### Induction of PmpD-N-specific antibodies in rHVT-*pmpD*-N vaccinated chickens

**Fig 3:**
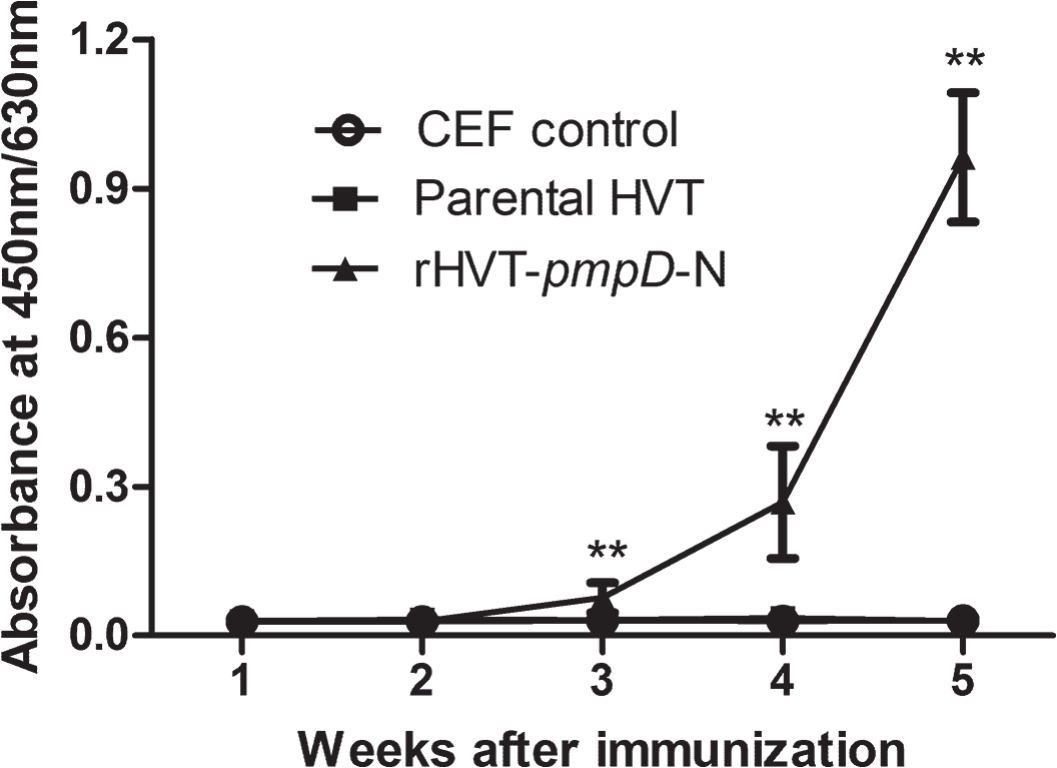
Humoral immune responses. Sera from CEF control group (n = 10), parental HVT group (n = 10) and rHVT-*pmpD*-N group (n = 15) were analyzed by ELISA assay for anti-PmpD-N antibody using plates coated with PmpD-N as the antigens as described in material and method section. Results are expressed as absorbance at 450nm/630nm. A serum sample was considered positive when its OD value exceeded 0.045. The values were shown as means ± standard deviation. ** Indicates *P* < 0.01 when rHVT-*pmpD*-N & parental HVT group or CEF control group.

Antibody levels in one-day-old chickens immunized with rHVT-*pmpD*-N were measured by an indirect ELISA in serum samples collected at weeks 1, 2, 3, 4 and 5 post-vaccination. Results showed that chickens were not seropositive until 3 weeks post vaccination (Fig 3), while antibody levels in 14 chickens in the rHVT-*pmpD*-N group were above the cut-off value at this time. All chickens were seropositive on day 28. The average OD values reached 0.96 on day 35. OD values of the CEF control group and parental HVT group was under the cut-off value at any time.

### Lymphocyte proliferation

**Fig 4:**
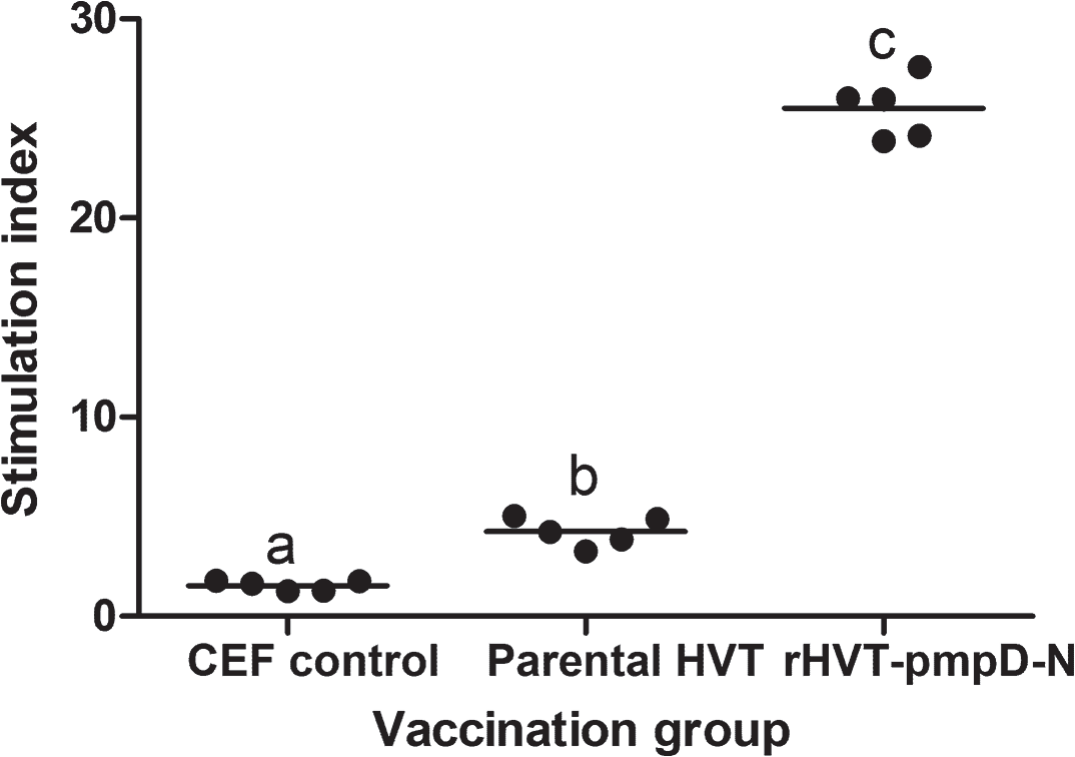
Antigen-specific proliferation responses. PBLs (*n* = 5 for each group) were obtained from immunized chickens on days 35, and lymphocytes were stimulated with 400 PFU of inactivated rHVT-*pmpD*-N. The results were examined as the stimulation index, calculated as the mean counts per minute (cpm) values of stimulated and non-stimulated wells. The values were shown as dot plots and means. ^a–c^Bars with the different superscripts indicate statistically significant difference among three groups.

Before challenge, proliferative responses of PBLs to rHVT-*pmpD*-N of chickens in the three groups were determined. In comparison with the CEF control group, both rHVT-*pmpD*-N group and parental HVT group induced significantly higher proliferative responses (*P*<0.05) (Fig 4). The stimulation index of group rHVT-*pmpD*-N vaccine was significantly higher than that of group parental HVT (*P*<0.05) (Fig 4), suggesting a possible effect of rHVT-*pmpD*-N.

### Flow cytometric analysis of T-lymphocyte subsets in peripheral blood

**Fig 5:**
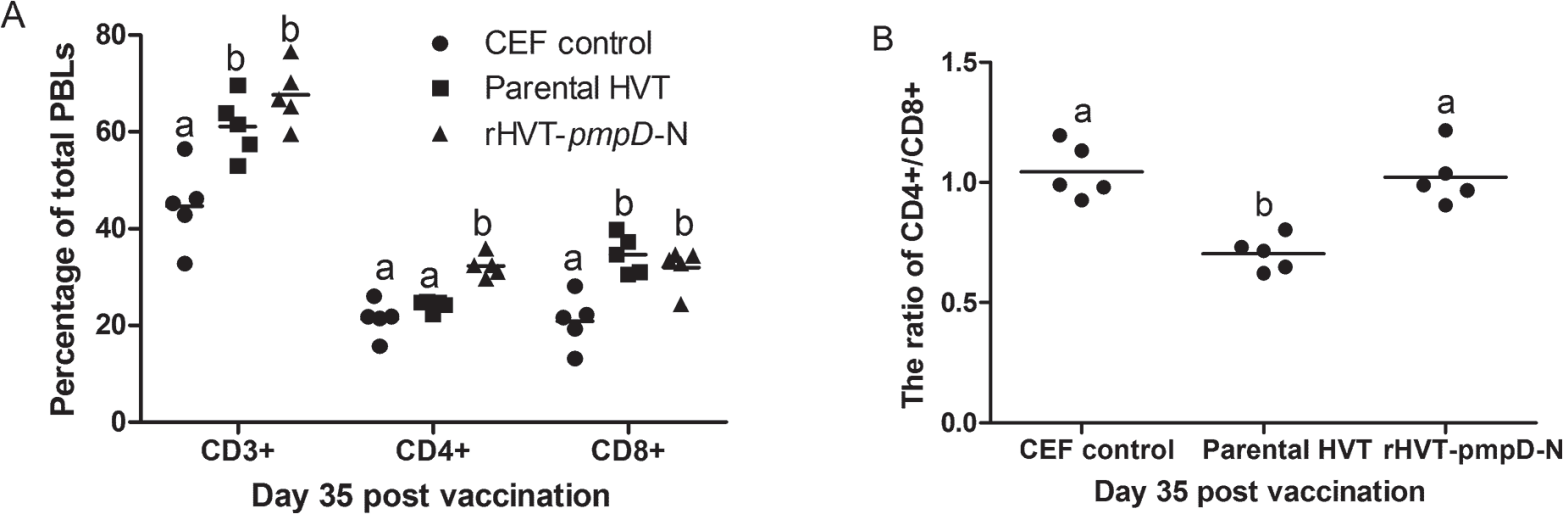
T lymphocyte subsets induced by immunization with rHVT-*pmpD*-N and parental HVT. T cells were purified from the blood of immunized chickens (*n* = 5 for each group) on day 35. The values were shown as dot plots and means. (A) The proportion of T cell subsets in PBLs by flow cytometric analysis. ^a–b^Bars in the same T lymphocyte subsets with the different superscripts indicate significantly different among three groups. (B) The ratio of CD4+/CD8+ among three groups. ^a–b^Bars with the different superscripts indicate statistically significant difference among three groups.

Before challenge, CD3+, CD4+ and CD8+ T-cell subsets were identified by flow cytometry, staining the T-cell subpopulations by use of monoclonal cell surface markers (Fig 5). A significantly higher mean percentage of CD3+ and CD8+ was found in rHVT-*pmpD*-N or HVT groups as compared to the CEF control group (*P*<0.05). The proportion of CD4+ in rHVT-*pmpD*-N group was significantly higher than that in other groups (P < 0.05). However, no statistical difference was found in the proportion of CD3+ and CD8+ between the rHVT-*pmpD*-N vaccine and parental HVT groups.

### Protection against *C*. *psittaci* challenge

**Fig 6:**
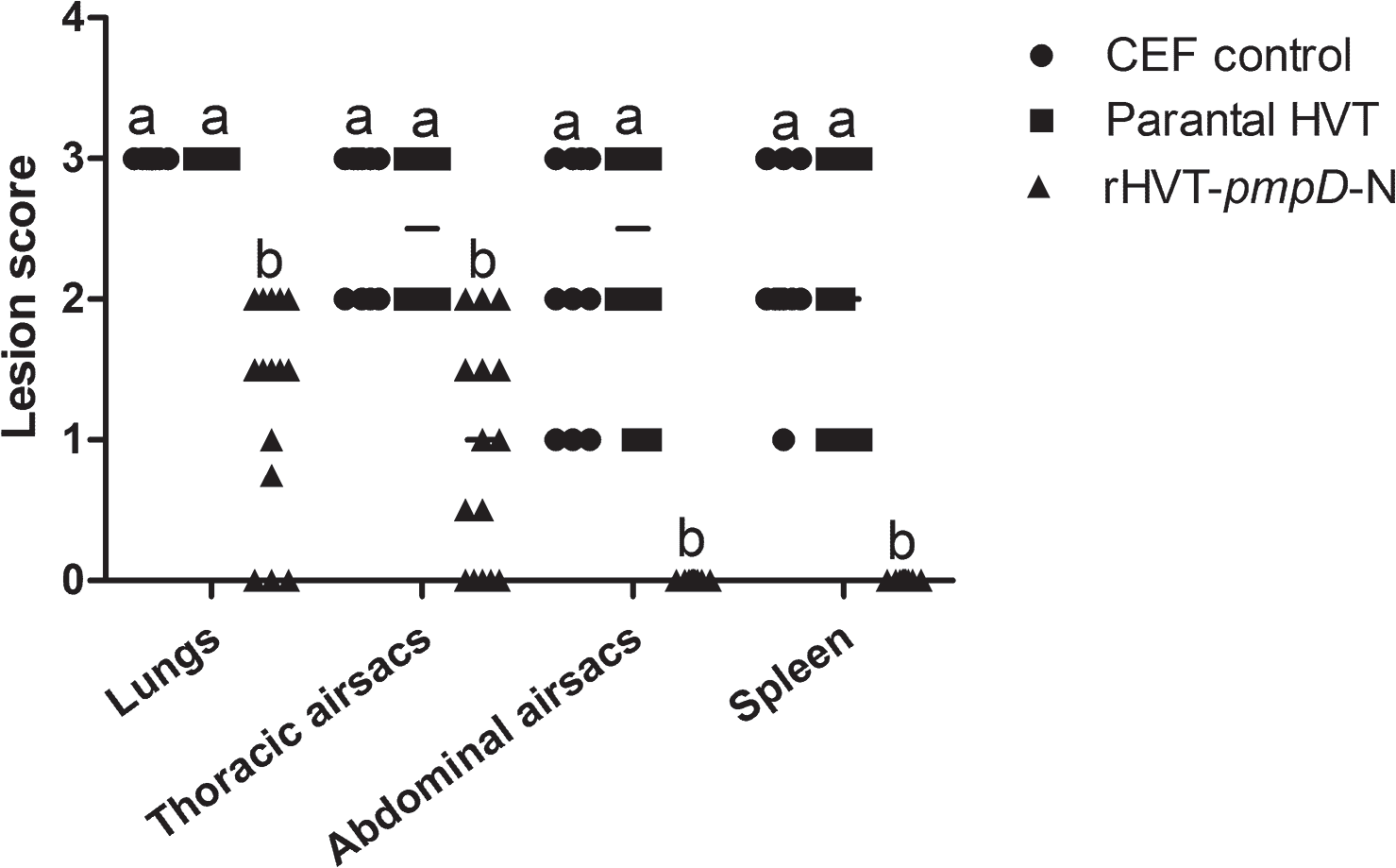
Lesion scores in chickens after challenge. Gross lesions (*n* = 10 for CEF control group and parental HVT group, *n* = 15 for rHVT-*pmpD*-N group) in target organs were assessed, and higher scores represent more severe lesions. The values were shown as dot plots and medians. ^a–b^Bars in the same tissue with the different superscripts indicate significantly different among three groups.

At euthanasia, all groups were examined for gross lesions. All chickens in the CEF and parental HVT groups showed severe lesions in the lungs, airsacs and spleens. In the rHVT-*pmpD*-N group, no lesions were observed in the lungs of 20% chickens (3/15) and thoracic airsacs of 26.7% chickens (4/15). The average gross lesion scores of lungs or thoracic airsacs of chickens in the rHVT-*pmpD*-N group were significantly lower than those of other groups (Fig 6). No apparent histopathological lesions in abdominal airsacs and spleens were observed in the rHVT-*pmpD*-N group (Fig 6). Therefore, good protection was observed in the chickens vaccinated with rHVT-*pmpD*-N.

**Fig 7:**
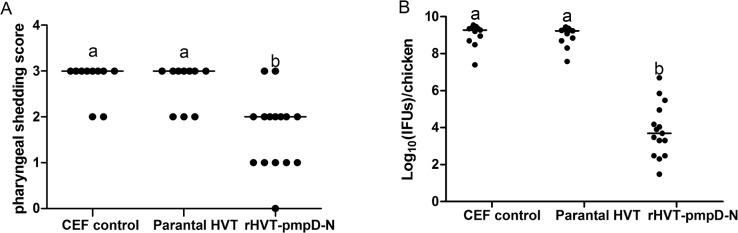
Pharyngeal shedding of challenge strain and chlamydial loads in lungs after challenge. The number of chlamydial inclusions in each group (*n* = 10 for CEF control group and parental HVT group, *n* = 15 for rHVT-*pmpD*-N group) was counted in five randomly selected microscopic fields as described in M&M section. The values were shown as dot plots and medians. (A) Median score of pharyngeal shedding of *C*. *psittaci* CB7 strain. (B) Chlamydial loads in lungs after challenge. ^a–b^Bars in the lungs or pharyngeal swabs with the different superscripts are significantly different.

With respect to *Chlamydia* shedding and clearance, the pharyngeal swabs were used to examine the excretion of *C*. *psittaci* using culture in BGM cells. Chickens in the CEF and parental HVT groups had high excretion scores (Fig 7A). Immune protection was evident in the rHVT-*pmpD*-N group which had significant lower pharyngeal excretion measurements than the other groups (Fig 7A).

Culture of lung suspensions revealed lower chlamydial replication in the rHVT-*pmpD*-N group compared to other groups, as the rHVT-*pmpD*-N group had significantly less inclusion-forming units (IFUs) than the other groups (Fig 7B).

## Discussion

Avian chlamydiosis is not only a health risk to chicken flocks, but also a threat to human health. Significant research is dedicated worldwide to curb the prevalence of *C*. *psittaci* in birds. The ultimate goal of *Chlamydia* vaccine research is to identify protective vaccine delivery methods that are capable of inducing the necessary protective immune response. Ideally, suitable vaccines should also elicit sterilizing immunity, limit the shedding of infectious organisms and the spread of infection [36]. Central to this objective is the need to develop safe delivery vehicles and adjuvants for optimal antigen presentation. HVT has proved to be a potent viral vector for the production of polyvalent vaccines that could provide effective protection against poultry diseases [24]. The recently described vaccine Vaxxitek^R^ HVT+ IBD has illustrated a promising application of the recombinant HVT vaccine approach [37]. In this study, an rHVT-*pmpD*-N plasmid derivative was shown to stably express the immunogenic protein fragment PmpD-N of *C*. *psittaci* in 20 passages in primary cell culture. Post immunization in chickens, it induced activated lymphocytes especially CD4+ subsets, which were associated with the reduced lesions and a significant decrease in chlamydial loads in the target organs post challenge with *C*. *psittaci* as compared to those of HVT vector or mock-infected control birds.

HVT is widely used as a live vaccine against Marek’s disease because of its antigenic relationship with MDV. In this study, we demonstrated that the growth kinetics of a recombinant HVT expressing chlamydial antigens was identical to that of the parental HVT. The result suggested that the insertion of the *pmpD*-N gene did not cause a profound effect on the infectivity and replicative ability of the recombinant rHVT-*pmpD*-N virus. Previous work has shown the recombinant HVT can confer a 100% protection against MDV challenge [24]. The MDV challenge experiment in this current study also showed the recombinant HVT could confer 100% protection against challenge with a very virulent MDV strain, which indicated the recombinant HVT kept the same immunological activity as parental HVT. Therefore, HVT-based delivery of a vaccine consisting of PmpD-N was able to induce robust immunity and protective efficacy against both MD and avian chlamydiosis with only one immunization.

In the current study, both the PmpD-N antigen and HVT vector might contribute to the protective immunity. Originally, two proteins on surface of *C*. *psittaci* were considered for use as protective antigens, including MOMP and PmpD. The 5'-terminus of the *pmpD* prokaryotic expression product was confirmed to induce a better immune response than recombinant MOMP in SPF chickens, suggesting that PmpD-N was a more promising vaccine candidate [21]. However, our previous experiment showed that only partial protection was achieved following two doses of the recombinant PmpD-N, with high lesion scores detected in lungs and spleens of vaccinated birds after challenge [21]. Ideally, an optimal delivery system should contribute to the cellular responses toward chlamydial clearance. Previous studies have shown that a MOMP-based DNA vaccine could not provide complete protection and could not be widely used in the poultry industry although the vaccine reduced severe clinical signs and bacterial replication in turkey models [14–16]. However, a recombinant adenovirus vaccine expressing MOMP was shown to elicit high antibody levels, cellular immunity and good protection in chickens owing to the delivery of MOMP antigen from cell to cell [9]. Furthermore, a phage vaccine based on the *ompA* gene of *C*. *abortus* was able to induce a significant immune response in piglets [34]. Based on this evidence, both the identity of the selected vaccine candidate and optimization of the system of delivery are key to the development of an effective *Chlamydia* vaccine. Recently, the full-length genome of HVT vaccine strain FC-126 as a bacterial artificial chromosomes (BAC) was manipulated to allow construction of recombinant HVT expressing genes of avian pathogens [24]. This system allowed the hemagglutinin gene of H7N1 subtype avian influenza virus to be introduced into the nonessential region of the HVT genome (UL45 and UL46) by homologous recombination [24]. In our study, the expression of rHVT-*pmpD*-N using BAC system resulted in PmpD-N expression in the cytoplasm and on the cell surface, suggestive of its capability to elicit a protective immune response as a live vaccine. In previous reports, CD4+ and CD8+ were shown to be important T lymphocyte markers and the major histocompatibility complex (MHC) class II-restricted CD4+ T cells were essential to resolve the primary infection [38]. Meanwhile, the protective role of MHC class I-restricted CD8+ T cells was dispensable for protective immunity [38]. In our study, a significant increase of CD4+ T cell subsets was found in the rHVT-*pmpD*-N-vaccinated chickens, which was critical for protection against *C*. *psittaci* infection. Thus, the HVT-based delivery system appeared to contribute advantageously toward an elevated cellular response. Taken together, these observations indicate that the HVT delivery system is reliable and efficacious as a basis for the development of a chlamydial vaccine.

Vaccine efficacy studies must be conducted using well-characterized challenge protocol. In our previous challenge studies, several strains were used to infect SPF chickens using different inoculation routes. Chickens inoculated intra-laryngeally with *C*. *psittaci* CB7 strain were observed to have the most obvious gross lesions 4 days after challenge, demonstrating the suitability of the challenge model for use in vaccine efficacy studies [28]. In the present study, birds inoculated with the rHVT-*pmpD*-N vaccine were protected against the homologous genotype A of *C*. *psittaci* CB7 strain. The ability of the vaccine to confer cross protection against the other five genotypes of *C*. *psittaci* are unknown, although the 43 kDa PmpD-N protein from genotype A has demonstrated serological cross-reactivity with sera from genotype A, B and D in our preliminary studies.

An apparent disadvantage of the rHVT-*pmpD*-N vaccine was found to be the slowly-rising specific-antibody levels post-immunization when compared to recombinant adenovirus vaccines or a recombinant PmpD-N subunit vaccine. The presence of specific antibody was not detected until 3 weeks post vaccination and reached a high titer after 5 weeks in our study. In contrast, antibody levels were found to increase after 2 weeks and reached a peak after 3 weeks by using the recombinant adenovirus vaccines. This relatively poor response might be associated with the slow replication rate of HVT *in vivo* [39]. This shortcoming could be overcome by injection of the vaccine into 18-day-old embryonated chicken eggs, allowing the early replication *in ovo*. Further studies involving additional immunizations and optimization of the route of immunization may be needed to induce a most robust humoral response.

In summary, chickens immunized with an rHVT-*pmpD*-N vaccine displayed not only high resistance to MDV infection, but also a good level of protection against *C*. *psittaci* disease with less lesions and reduced chlamydial loads. The rHVT-*pmpD*-N vaccine appears to act as a polyvalent immunization regimen against both MDV challenge as well as avian *C*. *psittaci* infection.

## Supporting Information

S1 Dataset Generation of 6BC-specific polyclonal antibodies and mouse anti-PmpD-N polyclonal serum of *C*. *psittaci*. (DOC)

S1 Fig Cytopathic effect of parental HVT and rHVT-*pmpD*-N on CEF cells. (A) Morphology of the infected CEF cells induced by parental HVT or rHVT-*pmpD*-N (magnifications 100 ×). (B) Immunohistochemical staining of CEF cells post inoculation with parental HVT or rHVT-*pmpD*-N (magnifications 100 ×).
(TIF)

S2 Fig Genetic stability analysis of rHVT-*pmpD*-N was evaluated by the *pmpD*-N specific primer amplification. M, DL2000 ladder; Lane 1, CEF negative control; lane 2, the 3^rd^ passage of rHVT-*pmpD*-N; lane 3, the 5^th^ passage of rHVT-*pmpD*-N; lane 4, the 10^th^ passage of rHVT-*pmpD*-N; lane 5, the 15^th^ passage of rHVT-*pmpD*-N; lane 6, the 20^th^ passage of rHVT-*pmpD*-N.
(TIF)

S3 Fig Protein stability analysis of rHVT-*pmpD*-N was evaluated by indirect immunofluorescence. CEF cells were infected with rHVT-*pmpD*-N of different passages, incubated with mouse anti-PmpD-N polyclonal serum of *C*. *psittaci*, and then reacted with goat anti-mouse IgG labelled with Alexa Fluor 488 (green fluorescence). A, the 5^th^ passage of rHVT-*pmpD*-N infected cells; B, the 10^th^ passage of rHVT-*pmpD*-N infected cells; C, the 15^th^ passage of rHVT-*pmpD*-N infected cells; D, the 20^th^ passage of rHVT-*pmpD*-N infected cells.
(TIF)
