# Supplementary material for: Construction of Recombinant HVT Expressing PmpD, and Immunological Evaluation against Chlamydia psittaci and Marek’s Disease Virus
Source: PLoS One. 2015 Apr 20;10(4):e0124992. doi: 10.1371/journal.pone.0124992 (PMC4404326; doi:10.1371/journal.pone.0124992)
Supplement: S1 Dataset — (DOC) [file pone.0124992.s001.doc]

S1_Dataset

**Text A. Generation of 6BC-specific polyclonal antibodies:**

SPF chickens were immunized subcutaneously with 1mg inactivated *C. psittaci* strain 6BC in complete Freund's adjuvant (Sigma-Aldrich, Shanghai, China) on day 0 and the same amount in incomplete Freund's adjuvant (Sigma-Aldrich, Shanghai, China) on the 14^th^, 21^st^, 28^th^ days following the first immunization. Seven days after final immunization, the sera of the immunized chickens were harvested. The titers of the anti-sera were 1:256 screened using IHA kit (LanzhouVeterinary Research Institute, Lanzhou, China).

**Text B. Generation of mouse anti-PmpD-N polyclonal serum of *C. psittaci*:**

BALB/C mice were immunized subcutaneously with 50 µg of purified PmpD-N protein in complete Freund's adjuvant on day 0 and with 100µg of purified PmpD-N protein in incomplete Freund's adjuvant on day 14^th^, 26^th^, 34^th^ following the first immunization. Five days after final immunization, the sera of the immunized mouse were harvested. The titers of the anti-sera were 1:100000 screened by ELISA.
