# Supplementary figures and images for: Construction of Recombinant HVT Expressing PmpD, and Immunological Evaluation against Chlamydia psittaci and Marek’s Disease Virus
Source: PLoS One. 2015 Apr 20;10(4):e0124992. doi: 10.1371/journal.pone.0124992 (PMC4404326; doi:10.1371/journal.pone.0124992)

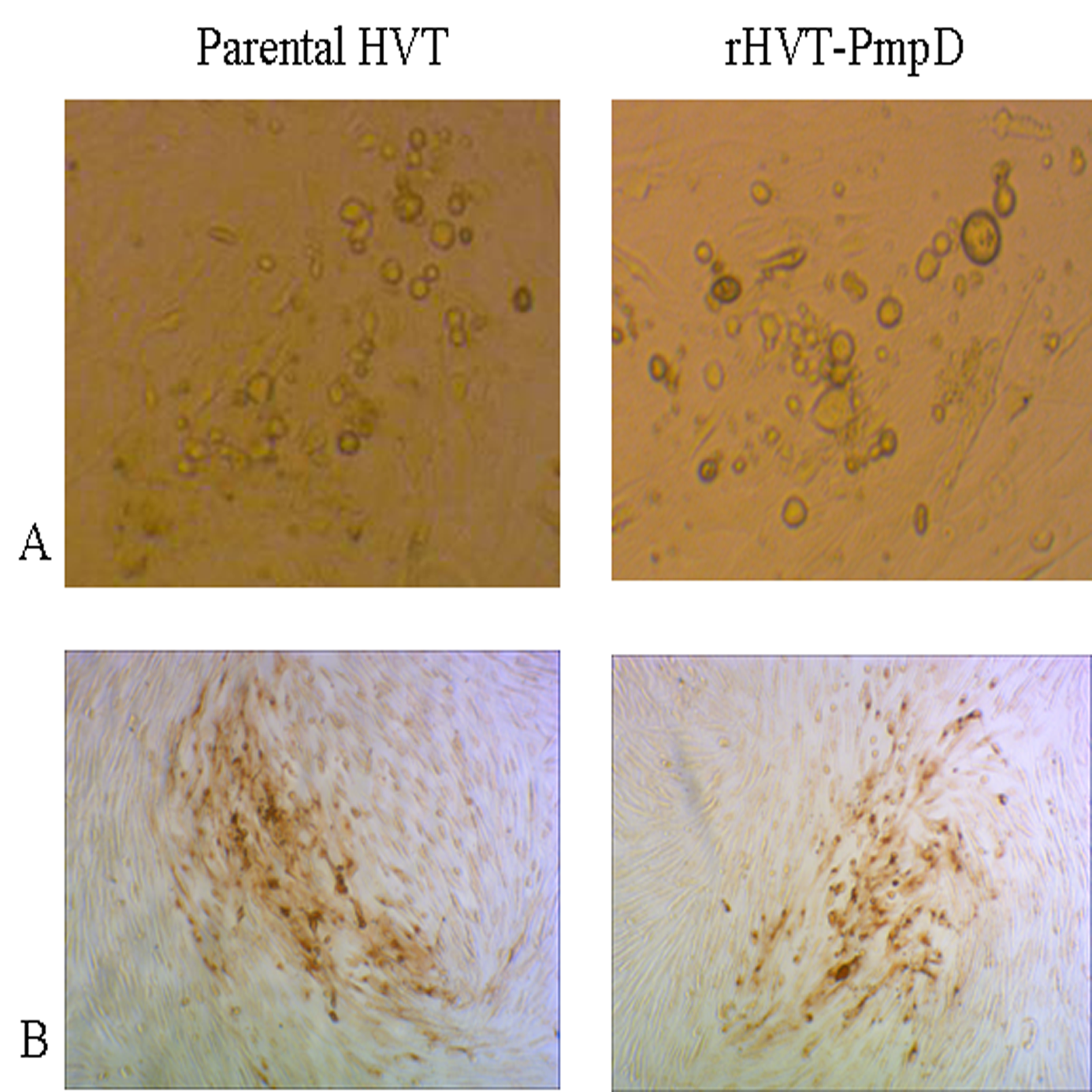

Supplement: S1 Fig — (A) Morphology of the infected CEF cells induced by parental HVT or rHVT-pmpD-N (magnifications 100 ×). (B) Immunohistochemical staining of CEF cells post inoculation with parental HVT or rHVT-pmpD-N (magnifications 100 ×). (TIF) [file pone.0124992.s002.tif]

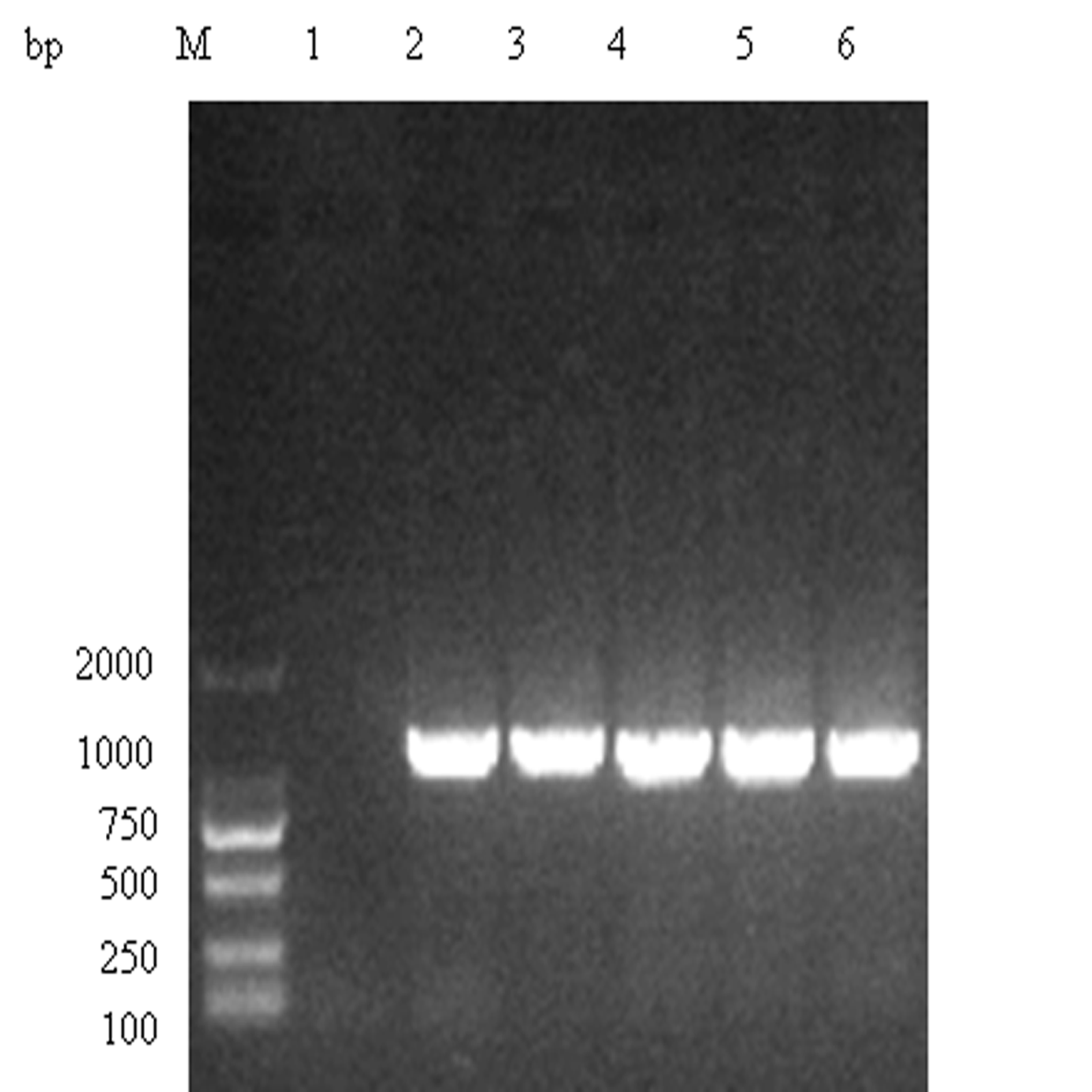

Supplement: S2 Fig — M, DL2000 ladder; Lane 1, CEF negative control; lane 2, the 3rd passage of rHVT-pmpD-N; lane 3, the 5th passage of rHVT-pmpD-N; lane 4, the 10th passage of rHVT-pmpD-N; lane 5, the 15th passage of rHVT-pmpD-N; lane 6, the 20th passage of rHVT-pmpD-N. (TIF) [file pone.0124992.s003.tif]

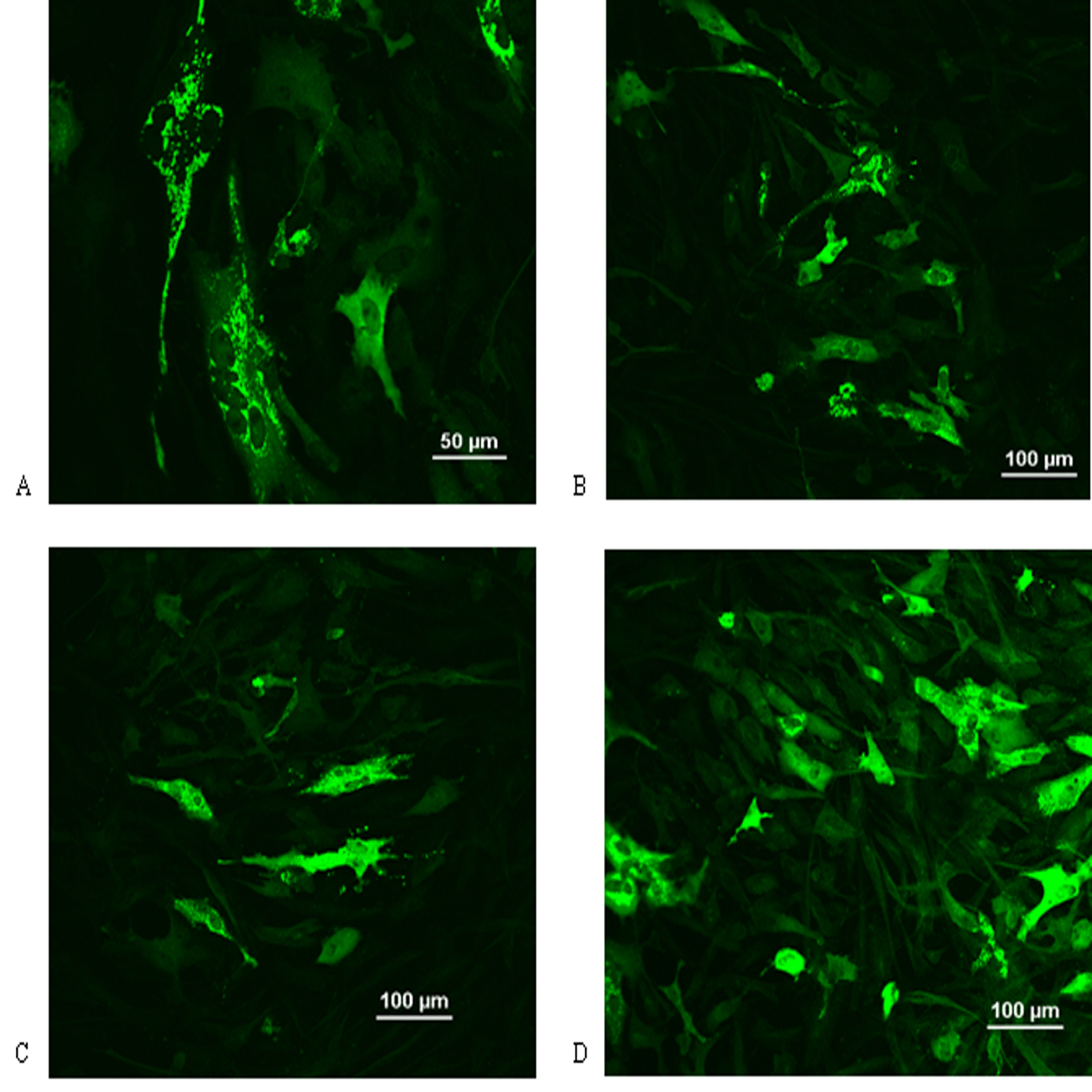

Supplement: S3 Fig — CEF cells were infected with rHVT-pmpD-N of different passages, incubated with mouse anti-PmpD-N polyclonal serum of C. psittaci, and then reacted with goat anti-mouse IgG labelled with Alexa Fluor 488 (green fluorescence). A, the 5th passage of rHVT-pmpD-N infected cells; B, the 10th passage of rHVT-pmpD-N infected cells; C, the 15th passage of rHVT-pmpD-N infected cells; D, the 20th passage of rHVT-pmpD-N infected cells. (TIF) [file pone.0124992.s004.tif]
